# Phylogeny and chronology of the major lineages of New World hystricognath rodents: insights on the biogeography of the Eocene/Oligocene arrival of mammals in South America

**DOI:** 10.1186/1756-0500-6-160

**Published:** 2013-04-22

**Authors:** Carolina M Voloch, Julio F Vilela, Leticia Loss-Oliveira, Carlos G Schrago

**Affiliations:** 1 Departamento de Genética, A2-097, Instituto de Biologia, Universidade Federal do Rio de Janeiro, Rua Prof. Rodolpho Paulo Rocco, SN Ilha do Fundão, Rio de Janeiro, CEP: 21941-617, Brazil; 2 Departamento de Genética, A2-095, Instituto de Biologia, Universidade Federal do Rio de Janeiro, Rua Prof. Rodolpho Paulo Rocco, SN Ilha do Fundão, Rio de Janeiro, CEP: 21941-617, Brazil; 3 Departamento de Genética, A2-092, Instituto de Biologia, Universidade Federal do Rio de Janeiro, Rua Prof. Rodolpho Paulo Rocco, SN Ilha do Fundão, Rio de Janeiro, CEP: 21941-617, Brazil

**Keywords:** Caviomorpha, Phiomorpha, Platyrrhini, Mitochondrial genome, Supermatrix, Bayesian relaxed clock

## Abstract

**Background:**

The hystricognath rodents of the New World, the Caviomorpha, are a diverse lineage with a long evolutionary history, and their representation in South American fossil record begins with their occurrence in Eocene deposits from Peru. Debates regarding the origin and diversification of this group represent longstanding issues in mammalian evolution because early hystricognaths, as well as Platyrrhini primates, appeared when South American was an isolated landmass, which raised the possibility of a synchronous arrival of these mammalian groups. Thus, an immediate biogeographic problem is posed by the study of caviomorph origins. This problem has motivated the analysis of hystricognath evolution with molecular dating techniques that relied essentially on nuclear data. However, questions remain about the phylogeny and chronology of the major caviomorph lineages. To enhance the understanding of the evolution of the Hystricognathi in the New World, we sequenced new mitochondrial genomes of caviomorphs and performed a combined analysis with nuclear genes.

**Results:**

Our analysis supports the existence of two major caviomorph lineages: the (Chinchilloidea + Octodontoidea) and the (Cavioidea + Erethizontoidea), which diverged in the late Eocene. The Caviomorpha/phiomorph divergence also occurred at approximately 43 Ma. We inferred that all family-level divergences of New World hystricognaths occurred in the early Miocene.

**Conclusion:**

The molecular estimates presented in this study, inferred from the combined analysis of mitochondrial genomes and nuclear data, are in complete agreement with the recently proposed paleontological scenario of Caviomorpha evolution. A comparison with recent studies on New World primate diversification indicate that although the hypothesis that both lineages arrived synchronously in the Neotropics cannot be discarded, the times elapsed since the most recent common ancestor of the extant representatives of both groups are different.

## Background

New World Hystricognathi (NWH, Caviomorpha) consists of a diverse assemblage of rodents that represent a unique level of ecological and morphological diversification among extant Rodentia. In size, caviomorphs vary from the largest living rodent, the capybara (*Hydrochoerus*), to the tiny degus (*Octodon*). The species in the lineage have exploited habitats as different as those used by the fossorial tuco-tuco (*Ctenomys*), the arboreal spiny rats (Echimyidae), the grazers such as the mara (*Dolichotis*) and the semi-aquatic capybara. Even representative species that were domesticated by humans, such as the chinchilla and the widely known guinea pig (*Cavia*), are found among NWH.

Despite their morphological and ecological diversity in the Neotropics, hystricognaths are not members of the endemic South American mammalian fauna. As didactically characterized by Simpson [1], caviomorphs, together with New World Primates (NWP, Platyrrhini), are part of the second major stage of South American mammal evolution [2]. They reached the New World during the Eocene, most likely by a transatlantic route from Africa [3]. This scenario is supported by the phylogenetic affinity of NWH with African hystricognath rodents (phiomorphs), particularly the families Thryonomyidae, Petromuridae and Bathyergidae [4]. Furthermore, the earliest record of caviomorphs in the New World is dated at approximately 41 Ma [5], when the South American continent was an isolated landmass.

Because of the evident biogeographical appeal of the topic, the evolution of Caviomorpha has motivated several studies that estimated divergence times, especially those using relaxed molecular clock techniques, to obtain a precise timescale for the origin of NWH [6-9]. Moreover, the close association of NWH evolutionary history with the origin of Neotropical primates, which also evolved from African ancestors that reached South America during the Eocene, has encouraged the comparative analysis of the problem [10,11].

The ages of the diversification events within NWH, however, have garnered comparatively less attention than the age of the separation of the Caviomorpha from African phiomorphs. Paleontological findings support the hypothesis that the diversification of caviomorphs consisted of a rapid event because the majority of the extant families were already present in the fossil record of the Deseadan (from 29 to 21 Ma, late Oligocene/early Miocene) [12]. Thus, if the earliest NWH fossils have an age of 41 Ma, the radiation of extant caviomorph families occurred approximately from the late Eocene to late Oligocene interval. This history indicates that the early divergences that produced supra-familial groupings may have occurred soon after the arrival of the ancestral stock.

In addition, there remain unresolved issues related to NWH macroevolution. Although the four major caviomorph lineages, the Cavioidea, Chinchilloidea, Erethizontoidea and Octodondoidea, which were ascribed to superfamilies by Woods [13], have been recovered in molecular phylogenetic analyses [4,8], the evolutionary affinities among these lineages are not consensual. For example, the first analyses based on molecular data identified the Erethizontoidea as the sister lineage of the (Chinchilloidea + Octodontoidea) clade and indicated the exclusion of the Cavioidea as a sister to all extant caviomorph superfamilies [4,6]. Recently, however, based on the analysis of additional genes, it appears that NWH consists of two major evolutionary lineages, the (Chinchilloidea + Octodontoidea) and the (Erethizonthoidea + Cavioidea) [7,14], although Rowe et al. [8] could not assign the Erethizontoidea to either the Cavioidea or the (Chinchilloidea + Octodontoidea) clade with statistical support.

Therefore, the early evolution of NWH raises issues that require further investigation to allow a deeper understanding of the geoclimatic factors that acted on the history of the group. Accordingly, the phylogenetic relationships among caviomorph superfamilies and the chronological setting in which the early diversification occurred are fundamental information for proposing consistent hypotheses about NWH origins. To achieve this goal, molecular data have been used successfully over the past decade. In this study, we increased the amount of mitochondrial data by sequencing the mitochondrial genomes of *Chinchilla lanigera* (Chinchilloidea), *Trinomys dimidiatus* (Octodontoidea) and *Sphiggurus insidiosus* (Erethizontoidea). The choice of mitochondrial markers is based on the recognition that the majority of molecular studies on Caviomorpha relied fundamentally on nuclear genes. Previous studies have already sequenced mitochondrial genomes of cavioids [15] and other octodontoids [16]. Therefore, the mitochondrial genomes of all NWH superfamilies were sampled.

We show that the combined analysis of nuclear genes and mitochondrial genomes supports the association of Erethizontoidea with Cavioidea and the separation of these associated taxa from the (Chinchilloidea + Octodontoidea) clade. The diversification of Caviomorpha from the African phiomorphs occurred approximately 43 Ma, and the early evolution of the major lineages occurred in the late Eocene. In contrast, family-level divergences occurred in the early Miocene, as supported by fossil record of the caviomorphs.

## Results

The *Trinomys dimidiatus*, *Chinchilla lanigera* and *Sphiggurus insidiosus* mitochondrial genomes were 16,533 bp, 16,580 bp and 16,571 bp long, respectively. The genomes presented the same gene order found in other mammals. The observed base frequencies were: *f*_A_ = 33.4%, *f*_C_ = 25.4%, *f*_G_ =13.5% and *f*_T_ = 27.7%, in the *T. dimidiatus* mitochondrial genome. In the *C. lanigera* mitochondrial genome the values were: *f*_A_ = 33.4%, *f*_C_ = 27.8%, *f*_G_ =13.1% and *f*_T_ = 25.8%. Finally, in the *S. insidiosus* mitochondrial genome, the base frequencies were: *f*_A_ = 33.5%, *f*_C_ = 22.7%, *f*_G_ =12.5% and *f*_T_ = 31.2%. These values are close to the average base frequencies estimated from the previously available hystricognath mitogenomes (*f*_A_ = 31.9%, *f*_C_ = 25.2%, *f*_G_ =12.3% and *f*_T_ = 30.7%).

**Figure 1:**
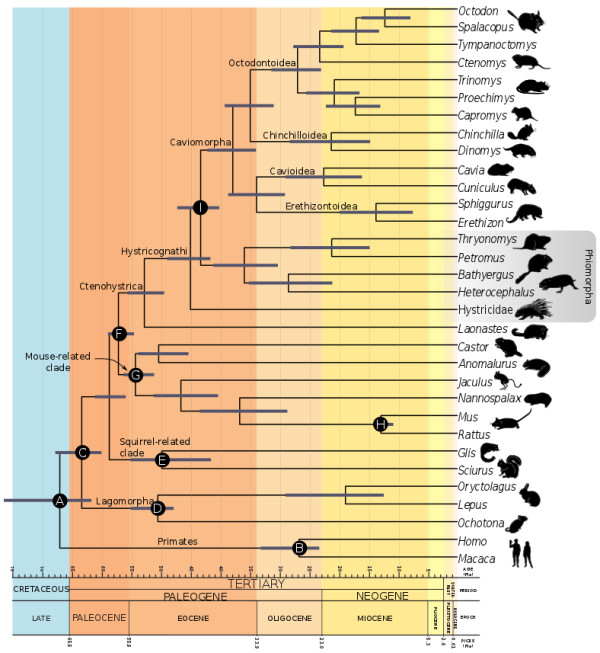
**Timescale for hystricognath evolution.** Statistical support for all nodes is 100% BP, except for the *Capromys + Proechimys* association, which is supported by 68% BP. Bars on nodes indicate the 95% credibility interval. Letters on nodes indicate the calibration information used.

All nodes of the inferred phylogeny were supported by 100% Bayesian posterior clade probability (BP), except for the divergence within the Echimyidae. The separation of Rodentia and Lagomorpha was estimated to have occurred at 63.4 Ma (Figure 1). The first rodent offshoot was composed of the Sciuromorpha. This event was inferred to have occurred at 58.8 Ma, in the late Paleocene. The split of the Hystrocognathi from other rodent lineages was also estimated in the late Paleocene, at 57.2 Ma (100% BP). The diversification of the *Castor*/*Anomalurus* lineage from myomorph rodents was inferred to have occurred in the early Eocene, at 54.4 Ma. The Castorimorpha/Anomaluromorpha split was also estimated in the early Eocene (50.4 Ma). All other myomorph splits studied, with the exception of the *Mus*/*Rattus* separation, were also inferred to have occurred in the Eocene.

Within Hystricomorpha, the separation of the Diatomyidae, represented by *Laonastes*, from other hystricognath rodents was inferred in the early Eocene (52.8 Ma). We recovered the Phiomorpha as a paraphyletic assemblage, consisting of Hystricidae and a clade with the remaining, strictly African-distributed, phiomorphs. This basal split between phiomorphs was inferred at 45.1 Ma (late Eocene). The New World Hystricognathi was recovered as monophyletic and sister to the phiomorph clade distributed exclusively in Africa. The separation between Old World and New World Hystricognathi was estimated to have occurred at 43.3 Ma, in the middle Eocene.

The basalmost split within the Caviomorpha consisted of the separation of the (Cavioidea + Erethizontoidea) superfamilies from the (Chinchilloidea + Octodontoidea). This split age was estimated from the middle to late Eocene, at 37.9 Ma. The Chinchilloidea/Octodontoidea divergence was inferred at 35.0 Ma (late Eocene), while the Cavioidea/Erethizontoidea separation also was inferred to have occurred at the end of the Eocene epoch (33.9 Ma). The oldest separation was that between (Echimyidae + Capromyidae) and (Octodontidae + Ctenomyidae) lineages, within Octodontoidea, which age was estimated at 27 Ma (late Oligocene). Family-level cladogenetic events were estimated to took place in the early Miocene epoch. Within octodontoids, the Ctenomyidae and Octodontidae divergence was inferred at 23.4 Ma, and the Capromyidae separation from the paraphyletic Echimyidae was estimated to have occurred at 17.2 Ma. Echimyidae paraphyly is weakly supported because the (*Capromys* + *Proechimys*) BP was 68%. The age of the separation between Dinomyidae and Chinchillidae, within chinchilloids, was inferred as 21.3 Ma. In cavioids, the Cuniculidae and Caviidae likely diverged in the early Miocene at 22.6 Ma. Diversification at the genus level probably occurred from the middle to the late Miocene.

## Discussion

The chronology of NWH evolution inferred from the combined analysis of mitochondrial genomes and nuclear data is compatible with the paleontological scenario recently proposed by Antoine et al. (2012). Note that these authors have also suggested that the caviomorph-phiomorph separation occurred at approximately 43 Ma, which is identical to our estimate. Our results are also consistent with the latest molecular analyses [7,8,14]. Therefore, the general pattern of caviomorph evolution is replicated by different analytical approaches. This outcome suggests that a consensus may have been reached. It is worth noting that our timescale is also in agreement with the recent hystricognath fossil findings from the Yahuarango Formation in Peruvian Amazonia. The fauna recovered from this formation, which yielded the first caviomorph record in the New World, is composed of animals with fundamentally plesiomorphic tooth morphology that resembles the early Afro-Asian phiomorphs from the middle Eocene (Antoine et al. 2012). These animals therefore represent the early stages of NWH evolution and are most likely not directly related to any of the extant lineages. This hypothesis is consistent with our findings because the extant lineages diversified after 37.6 Ma according to our timescale.

In terms of the general pattern of diversification, the Caviomorpha evolved from an African hystricognath lineage in the middle Eocene. The extant (Thryonomyidae + Petromuridae), Bathyergidae) clade consists of its phiomorph sister group, excluding the living Hystricidae, which may descend from the first phiomorph radiation. This phylogenetic arrangement was first proposed by Huchon and Douzery [4]. Within Caviomorpha, the relationship between cavioids and erethizontoids is perhaps the most unusual hypothesis suggested by the molecular data. For example, McKenna and Bell [17] excluded the erethizontoids from the major Neotropical radiation of Hystricognathi, dubbed Caviida by those authors, which included octodontoids, chinchilloids and cavioids. In our study, the position of Erethizontoidea as the sister group of the Cavioidea is statistically supported and is consistent with previous analyses [7,14]. We used the KH [18] and SH tests [19] to evaluate the statistical significance of the difference in log-likelihoods between our hypothesis and that of an alternative phylogeny that placed Erethizontoids with the (Octodontoidea + Chinchilloidea) clade. Both tests rejected the null hypothesis that the log-likelihoods of both phylogenies are equal (*p* < 0.05) in favor of the topology inferred in this study.

In addition, our phylogenetic hypothesis also corroborates an African origin of Caviomorpha. The age of the separation of NWH from African phiomorphs (43.3 Ma) is also in agreement with previous studies based primarily on nuclear data. Because the diversification of the first Neotropical hystricognath lineages occurred at 37.6 Ma, the colonization of the South American island continent must have occurred at some time before the middle Eocene. If this conclusion is correct, a transatlantic dispersal route was used. It is possible that this dispersal occurred as a result of island hopping along an island corridor [20] or even by floating islands, which, at least for primates, is a possibility to be considered [21].

Although a general consensus has been reached on the African origin of NWH [8,14,22], it is worth mentioning that an alternative hypothesis for the origin of caviomorphs was proposed by A. E. Wood [23], who considered the North American “Franimorpha” the possible ancestral stock of South American hystrichognaths. This association was based on the putative hystricomorphous condition of North American Eocene species such as *Platypittamys*. However, this hypothesis was primarily questioned by René Lavocat [3,24], who supported an African origin of caviomorphs. Recently, Martin [25] showed that the enamel microstructure of caviomorph teeth is similar to that found in certain African phiomorphs. Moreover, it is now generally considered that franimorphs were actually protogomorph rodents, with no association with the radiation of caviomorphs [26].

As previously stated, because of the biogeographic importance of the problem, it is customary to perform studies on NWH evolution in conjunction with a comparative analysis of the evolution of the Neotropical primates. The latest extensive analysis of primate evolution, conducted by Perelman et al. [27], inferred that the New World Platyrrhini/Old World Catarrhini separation occurred at 43.5 Ma. This value is statistically identical to the age of the caviomorph-phiomorph split estimated here. These estimates agree with the recent analysis of Loss-Oliveira et al. [11] and the earlier proposal by Poux et al. [10], who showed that the available molecular data cannot reject the hypothesis of a synchronous arrival of hystricognaths and primates in the New World.

As noted by Antoine et al. [5], the evolutionary history of anthropoids and hystricognaths is curiously linked. Both groups are hypothesized to have evolved in Asia and then to have invaded Africa from the early to middle Eocene [28]. As molecular data suggest, the probability of a single colonization event involving the isolated South American continent is high. However, paleontological findings on primates support a later arrival of anthropoids [29]. The lag between the first hystricognaths and the first representative of the Platyrrhini, *Branisella* sp., is close to 15 Ma [5]. Because molecular data represent the time of genetic separation of lineages, it is possible that the Platyrrhini/Catarrhini divergence may not be associated with the dispersal event from Africa to South America. In this scenario, the genetic separation would have occurred on the African continent, with a subsequent dispersal of anthropoids to the Neotropics. This hypothesis would imply that fossil anthropoids with platyrrhine characteristics should occur in Africa. Actually, as Fleagle [30] reported, fossils recovered from the Eocene deposit of Fayum, Egypt, show certain NWP attributes. Nevertheless, these attributes may represent the plesiomorphic anthropoid morphology and would only indicate that NWP morphology remained plesiomorphic during its evolutionary history.

Another important issue is that, in contrast to the value for the Hystricognathi, the time to the most recent common ancestor of extant NWP is inferred to be ca. 20 Ma, in the early Miocene [27,31,32]. Therefore, the living NWP are the descendants of a younger stock than the caviomorphs. This finding implies that the pattern of lineage extinction was distinct in both groups. This topic has been investigated recently by Kay et al. [33], who proposed that *Branisella* and several NWP fossils from the Miocene deposits of the southern region of South America represent an independent radiation, not related to any of the extant Platyrrhini lineages. In caviomorphs, however, the early Oligocene record is already associated with one of the major extant lineages [5,34,35].

## Conclusion

In conclusion, the chronology of NWH evolution inferred from the combined analysis of nuclear genes and mitochondrial genomes indicates that Caviomorpha/phiomorph separation and the early diversification of NWH lineages in South America occurred in the middle Eocene. Extant caviomorphs are composed of two major lineages: the (Chinchilloidea + Octodontoidea) and (Cavioidea + Erethizontoidea). Family-level splits took place in the early Miocene epoch. Compared with New World primates, caviomorph lineages are older, but the hypothesis of a single colonization event cannot be discarded.

## Methods

Total genomic DNA was obtained from fresh or ethanol-preserved fragments of hepatic tissue from three specimens: *Trinomys dimidiatus* (the soft-spined Atlantic spiny-rat, field number JFV224, accession number JX312694), *Chinchilla lanigera* (the chinchilla, JFV368, accession number JX312692) and *Sphiggurus insidiosus* (the Bahia hairy dwarf porcupine, JFV386, accession number JX312693). Genomic DNA was extracted with QIAamp® DNA Mini and Blood Mini kit. DNA was quantified with a NanoDrop spectrophotometer. Paired-end sequencing was performed with the Illumina HiSeq 2000 platform by Fasteris (http://www.fasteris.com). The mitochondrial genome was de novo assembled using the CLC Genomics Workbench 5.1 program with default settings. Sample collection was performed following the national guidelines and provisions of IBAMA (Instituto Brasileiro do Meio Ambiente e dos Recursos Naturais Renováveis, Brazil), under permit number 109/2006. Therefore, all animal procedures were conducted under the jurisprudence of the Brazilian Ministry of Environment and its Ethical Committee. This study does not involve laboratory work on living animals.

### Evolutionary analysis

The species used in this study, as well as accession numbers, are listed in Table 1. In addition to NWH, we included representatives of several lineages of Glires and rooted the tree with primate outgroups. Mitochondrial genomes were analyzed by selecting the 13 protein-coding genes. We also studied six publicly available nuclear genes: ADRA2, IRBP, vWF, GHR, BRCA1 and RAG1. The genes were aligned individually in CLUSTALW [36] and then concatenated in a 22,548 bp supermatrix, all three codon positions were included in the matrix. Phylogenetic inference was conducted with MrBayes 3.2 [37] using the GTR + G4 + I model of sequence evolution, which was chosen by the likelihood ratio test implemented in HyPhy [38]. Two independent runs with four chains each (one cold and three hot chains) were sampled every 1,000^th^ generation until 10,000 trees were obtained. A burn-in of 1,000 trees was applied. Chain convergence was monitored by the standard deviation of split frequencies, which reached a plateau at 0.0004, and the potential scale reduction factor statistic, which approached 1.00 for all parameters.

**Table 1:** Accession numbers and taxonomic sampling used in this study

**Terminal**	**Species**	**ADRA2B**	**IRBP**	**vWF**	**GHR**	**BRCA1**	**RAG1**	**Mitochondrial genome**	**Cox1**	**Cytb**
*Mus*	*Mus musculus*	NM_009633	AF126968	U27810	BC075720	NM_009764	NM_009019	NC_005089		
*Rattus*	*Rattus norvegicus*	M32061	AJ429134	AJ224673	NM_017094	NM_012514	NM_053468	NC_001665		
*Nannospalax*	*Nannospalax ehrenbergi*	AM407905	JN414825	FM162064	AY294898	JN414208	JN414978	NC_005315		
*Jaculus*	*Jaculus jaculus*	AM407906	AM407907	AJ297765	AF332040	JN414198	JN414964	NC_005314		
*Glis*	*Glis glis*	AJ427258	AJ427235	AJ224668	AM407916		AB253971	NC_001892		
*Sciurus*	*Sciurus* sp.^1^	AJ315942	AY227620	AM407918	AF332032	AF332044	AY241477	NC_002369		
*Castor*	*Castor Canadensis*	AJ427260	AJ427239	AJ427228	AF332026	AF540622	JN414956	NC_015108		
*Anomalurus*	*Anomalurus* sp.^2^	AJ427259	AJ427230	AJ427229	AM407919	JN414191	JN414951	NC_009056		
*Laonastes*	*Laonastes aenigmamus*	AM407899	AM407903	AM407897	AM407901	JN414207	JN414977			AM407933
*Thryonomys*	*Thryonomys swinderianus*	AJ427267	AJ427243	AJ224674	AF332035	JN414206	JN414976	NC_002658		
*Petromus*	*Petromus typicus*	AJ427268	AJ427244	AJ251144	JN414761	AF540639	JN414974			DQ139935
*Bathyergus*	*Bathyergus suillus*	AJ427252	AJ427251	AJ238384	FJ855201					AY425913
*Heterocephalus*	*Heterocephalus glaber*	AM407924	AM407925	AJ251134	AF332034	AF540630	JN414953	NC_015112		
Hystricidae	*Trichys* sp./*Hystrix* sp.^3^	AJ427266	AJ427245	AJ224675	AF332033	AF540631	JN414970		JN714184	FJ472577
*Chinchilla*	*Chinchilla lanigera*	AJ427271	AJ427246	AJ238385	AF332036	JN414194	JN414958	**JX312692**		
*Dinomys*	*Dinomys branickii*	AM050859	AM050862	AJ251145	AF332038	DQ354450	JN414963			AY254884
*Cavia*	*Cavia porcellus*	AJ271336	AJ427248	AJ224663	AF238492		NT_176327	NC_000884		
*Cuniculus*	*Cuniculus* sp.^4^	AM050861	AM050864	AJ251136	AF433928	JN414190	JN414950		JF459149	AY206573
*Trinomys*	*Trinomys* sp.^5^			AJ849316			EU313337	**JX312694**		
*Proechimys*	*Proechimys* sp.^6^			AJ251139	AF332039		EU313332	HM544128		
*Capromys*	*Capromys pilorides*	AM407926	AM407927	AJ251142	AF433949	JN414192	JN414954			AF422915
*Tympanoctomys*	*Tympanoctomys barrera*				AF520655			HM544132		
*Spalacopus*	*Spalacopus cyanus*				AF520653			HM544133		
*Octodon*	*Octodon* sp.^7^	AM050860	AM050863	AJ238386	AM407928			HM544134		
*Ctenomys*	*Ctenomys* sp.^8^	JN413825	JN414816	JN415078	JN414757	JN414196	JN414961	HM544130		
*Sphiggurus*	*Sphiggurus* sp.^9^			AJ224664	FJ855212			**JX312693**		
*Erethizon*	*Erethizon dorsatum*	AJ427270	AJ427249	AJ251135	AF332037	DQ354451	JN414966		JF456594	FJ357428
*Oryctolagus*	*Oryctolagus cuniculus*	Y15946	Z11812	U31618	AF015252	DQ354452	M77666	NC_001913		
*Lepus*	*Lepus* sp.^10^	AJ427254	AJ427250	AJ224669	AF332016	AF284005		NC_004028		
*Ochotona*	*Ochotona princeps*	AJ427253	AY057832	AJ224672	AF332015	AF540635	JQ073183	NC_005358		
*Homo*	*Homo sapiens*	AF316895	J05253	M25851	X06562	NM_007294	NG_007528	NC_012920		
*Macaca*	*Macaca mulatta*	AM050852	AJ313476	AJ410302	U84589	NM_001114949	NW_001100721	NC_005943		

**Table Footnote:** (1) *Sciurus vulgaris* (adra2b, IRBP), *Sciurus aestuans* (vWF), *Sciurus niger* (GHR, BRCA1), *Sciurus ignitus* (RAG1), *Sciurus vulgaris* (mitochondrion, complete genome); (2) *Anomalurus* sp. (A2AB, irbp, vWF, ghr), *Anomalurus beecrofti* (BRCA1, RAG1), *Anomalurus* sp. (mitochondrion, complete genome); (3) *Trichys fasciculata* (A2AB, irbp, VWF), *Hystrix africaeaustralis* (GHR, BRCA1), *Hystrix brachyurus* (RAG1), *Hystrix indica* (CO1) *Hystrix cristata* (cytb); (4) *Cuniculus paca* (adra2b, irbp, vWF, GHR), *Cuniculus taczanowskii* (BRCA1, RAG1), *Cuniculus paca* (CO1, cytb); (5) *Trinomys paratus* (vWF), *Trinomys iheringi* (RAG1), *Trinomys dimidiatus* (mitochondrion, complete genome); (6) *Proechimys oris* (vWF), *Proechimys longicaudatus* (GHR), *Proechimys simonsi* (RAG1), *Proechimys longicaudatus* (mitochondrion, complete genome); (7) *Octodon lunatus* (adra2b, irbp, vWF), *Octodon degus* (ghr, mitochondrion, complete genome); (8) *Ctenomys boliviensis* (adra2b, IRBP, vWF, GHR, BRCA1, RAG1), *Ctenomys rionegrensis* (mitochondrion, partial genome); (9) *Sphiggurus melanurus* (vWF), *Sphiggurus mexicanus* (GHR), *Sphiggurus insidiosus* (mitochondrion, complete genome); (10) *Lepus crawshayi* (A2AB, irbp, vWF), *Lepus capensis* (GHR, BRCA1), *Lepus europaeus* (mitochondrion, complete genome).

Divergence time estimation was performed in the MCMCTree program of the PAML 4.5 package [39] with the multivariate normal approximation [40]. The model of evolutionary rate evolution adopted was the independent lognormal [41]; nucleotide substitutions were modeled by the HKY85 + G6, which is the parameter richer model implemented in MCMCTree. After a burn-in period of 50,000 generations, the Markov chain Monte Carlo (MCMC) algorithm was sampled every 100^th^ generation until 20,000 samples of divergence time parameters were obtained. Detailed prior information for the model parameters is as follows: BDparas = 1 1 0; kappa_gamma = 6 2; alpha_gamma = 1 1; rgene_gamma = 2 2 and sigma2_gamma = 1 10. Convergence of the MCMC runs was measured by the effective sample sizes and the potential scale reduction factor [42].

### Calibration information

We have used nine calibration priors to estimate the posterior density of divergence times (Figure 1): (A) The Primates/Glires split was constrained to have occurred between 100.5 and 61.5 Ma [43,44]; (B) Within the Primates, the *Homo*/*Macaca* separation was assigned a uniform prior from 34 to 23.5 Ma based on the fossil findings of *Proconsul* and *Catopithecus*[45,46]; (C) The Lagomorpha/Rodentia split was assigned a minimum age of 61.5 Ma based on the age of *Heomys*, an early rodent [43]; (D) Within Lagomorpha, the Leporidae/Ochotonidae split was constrained by a uniform distribution from 48.6 to 65.8 Ma based on the Vastan fossils [47]; (E) The separation of the Sciuromorpha (*Sciurus*/*Glis*) from the rest of the rodents was constrained to have occurred between 55.6 and 65.8 Ma based on *Sciuravus*[48]. (F) The split of Hystricognathi + *Laonastes* from myomorph and castorimorph rodents was assigned a uniform prior from 52.5 to 58.9 Ma based on *Birbalomys*, an early hystricognath [49]. (G) The separation of the *Castor*/*Anomalurus* lineage from other myomorph rodents was constrained by a uniform distribution from 56.0 to 40.2 Ma according to the fossil finding of *Ulkenulastomys*, an early myomorph [50]. (H) The *Mus*/*Rattus* split was enforced to have occurred between 10.4 and 14 Ma (*Karnimata*) [51]. (I) Finally, the Caviomorpha/“Phiomorpha” was assigned a minimum age of 40 Ma, based on the recent discoveries of hystricognath rodents from the Yahuarango Formation in Peru [5].

## Abbreviations

Ma: *Mega annum*

## Competing interests

Authors declare no competing interests.

## Authors’ contributions

CMV, JFV, LL-O and CGS carried out the molecular genetic studies, participated in the sequence alignment and drafted the manuscript. CMV and CGS participated in the design of the study and performed the statistical analysis. All authors read and approved the final manuscript.
